# Medium-term outcomes of total hip arthroplasty in juvenile patients

**DOI:** 10.1186/s13018-020-01990-2

**Published:** 2020-10-16

**Authors:** Francesco Luceri, Ilaria Morelli, Carlo Maria Sinicato, Alberto Della Grazia, Fabio Verdoni, Nicola Maffulli, Giuseppe M. Peretti, Domenico Curci

**Affiliations:** 1 IRCCS Istituto Ortopedico Galeazzi, 20161 Milan, Italy; 2 Residency Program in Orthopedics and Traumatology, University of Milan, Via Mangiagalli 31, 20133 Milan, Italy; 3 Casa di Cura I Cedri, HABILITA SPA, Via Bologna, 1, Zingonia di Ciserano, Bergamo, Italy; 4 U.O. Clinica Ortopedica e Traumatologica Universitaria CTO, Azienda Socio Sanitaria Territoriale Centro Specialistico Ortopedico Traumatologico Gaetano Pini-CTO, Piazza Cardinal Ferrari 1, 20122 Milan, Italy; 5 San Giovanni di Dio e Ruggi D’Aragona Hospital “Clinica Ortopedica” Department, University of Salerno, Salerno, Italy; 6 School of Pharmacy and Bioengineering, Keele University School of Medicine, Thornburrow Drive, Stoke on Trent, England; 7 Centre for Sports and Exercise Medicine, Barts and the London School of Medicine and Dentistry, Queen Mary University of London, Mile End Hospital, 275 Bancroft Road, London, E1 4DG England; 8 Department of Biomedical Sciences for Health, University of Milan, Via Mangiagalli 31, 20133 Milan, Italy

**Keywords:** Total hip arthroplasty, Paediatric, Juvenile hip osteoarthritis, Total hip replacement, Case series

## Abstract

**Background:**

Juvenile hip osteoarthritis is often the end result of congenital conditions or acquired hip ailments occurred during the paediatric age. This study evaluated the middle term results of total hip arthroplasty for end-stage juvenile hip osteoarthritis.

**Materials and methods:**

This is a retrospective analysis of prospectively collected data on a cohort of 10 consecutive patients (12 hips), aged between 14 and 20 at operation, who underwent cementless total hip arthroplasty for end-stage juvenile secondary hip osteoarthritis in two orthopaedic tertiary referral centres between 2009 and 2018.

**Results:**

Juvenile hip osteoarthritis occurred as a consequence of developmental dysplasia of the hip, Legg-Calvé-Perthes disease, femoral head necrosis or slipped capital femoral epiphysis. All patients showed a significant improvement in Harris Hip Score (*p* < 0.01) at 3.3 years average follow-up (range 0.7–10.1 years).

**Conclusion:**

The management of juvenile hip osteoarthritis following developmental dysplasia of the hip, Legg-Calvé-Perthes disease, femoral head necrosis or slipped capital femoral epiphysis is still challenging. Careful preoperative planning is essential to achieve good outcomes and improve the Harris Hip Score in these young patients. Total hip arthroplasty is a suitable option for end-stage secondary juvenile hip osteoarthritis, when proximal femoral osteotomies and conservative treatments fail to improve patients’ symptoms and quality of life.

**Level of evidence:**

IV

## Introduction

Osteoarthritis of the hip may be the end result of congenital or acquired hip conditions occurred during the juvenile age [1]. Developmental dysplasia of the hip (DDH), Legg-Calvé-Perthes disease (LCPD), slipped capital femoral epiphysis (SCFE) and hip joint infections are the most common cause of juvenile hip osteoarthritis (JHOA) [2]. The complex management and the catastrophic consequences of these conditions justify the active research interest in this field [3–6]. DDH ranges from mild dysplasia of the acetabulum to frank dislocation of the hip [7] and is one of the most common congenital deformities of the lower limb [8]. Up to 35% of DDH patients develop idiopathic avascular necrosis (AVN) 5 years after conservative treatment and up to 32.9% develop AVN 10 years after surgical treatment [9].

LCPD, with an incidence between 4 and 32 per 100,000 population per year, can be complicated by AVN [10]. The prognosis of LCPD is more favourable in patients with early onset regardless of treatment, but exceptions remain common [11]. Predicting which child will need a salvage procedure remains a major challenge, but approximately 5% of the affected children will require a total hip arthroplasty (THA) [12].

There seems to be an increased risk of hip osteonecrosis after systemic glucocorticoid administration in young patients [13]. Glucocorticoids negatively influence skeletal remodelling in children [14]. A strong association of AVN with high-dose glucocorticoid therapy has been reported in systemic diseases [15–17].

The complication rate after SCFE treatment is difficult to assess [18], given the lack of standardized clinical data reporting and multicentre studies. A recent study reported an overall 29.4% AVN rate in a cohort of patients with stable SCFE treated with modified Dunn procedure [2], compared to a cohort of patients with unstable slips, who experienced a 6% AVN rate [19]. A 4% incidence of anterolateral hip instability was also found after modified Dunn procedure [20].

Septic hip arthritis is managed by surgical drainage in patients younger than 10, open arthrotomy and lavage in older children [21] or hip arthroscopy [22]. The presentation of paediatric septic arthritis of the hip may be dramatic and sometimes needs a major surgery [23].

In most cases, the sequelae of paediatric hip abnormalities require THA in adulthood [24]. Nevertheless, when disability from end-stage JHOA compromise the daily living of these young patients, THA could be required in the paediatric age range [3, 25]. These patients and their parents do not usually accept a function-limiting option such as hip resection or arthrodesis [26, 27]. Furthermore, the adequate timing of this kind of surgery is controversial. The management of these paediatric conditions is particularly challenging because of the profound alterations in hip anatomy, sequelae of the previous surgery and limb length discrepancy [28].

Surface arthroplasty allows to preserve bone stock and could be easily converted to THA in case of implant failure [29]. Nevertheless, the difficult learning curve and the higher revision rate of surface arthroplasty make THA the treatment of choice in young patients [30–32]. The clinical outcome of THA in children, adolescents and young adults is largely unknown and difficult to evaluate [6].

This study evaluated the reliability of THA in the management of end-stage JHOA.

## Materials and methods

With appropriate Institutional Review Board approval, we retrospectively reviewed all patients affected by end-stage secondary JHOA who had undergone cementless THA between 2009 and 2018 at two major orthopaedic hospitals (Table 1). All patients were operated by two surgeons (C.M.S. and A.D.G.). For all patients, clinical features, hip pathologies leading to JHOA, prior surgeries, surgical approach for THA, implant type, surgical time, length of stay, and complications were recorded (Table 2). We also recorded the surgical approach used (Table 3).

**Table 1:** Study patients’ clinical data and Harris Hip Score

Patient	Gender	Age (years)	Hip	Pathology	Prior surgeries	HHS before THA	HHS after THA
1	F	18	Left	SCFE	Dunn procedure, hardware removal	35	93
2	F	16	Right	Glucocorticoid-induced osteonecrosis of femoral head in ALL	None	38	91
			Left			41	92
3	M	15	Left	SCFE	Dunn procedure, hardware removal	11.55	96
4	M	16	Left	SCFE	Dunn procedure, hardware removal	30	94
5	M	15	Right	SCFE	Screw fixation, hardware removal	58.4	96
6	M	14	Left	SCFE	Screw fixation, hardware removal	32	94
7	F	20	Left	LCPD	Arthrodiatasis of the hip with external fixator, hardware removal	26.55	95.5
8	M	17	Right	DDH	None	59	95.85
9	F	19	Left	DDH	Chiari’s pelvic osteotomy and hardware removal (left hip only)	25	92
		20	Right			36.5	90
10	M	18	Left	LCPD	None	40	94.5

*SCFE* slipped capital femoral epiphysis, *ALL* acute lymphoblastic leukaemia, *LCPD* Legg-Calvé-Perthes disease, *DDH* developmental dysplasia of the hip, *HHS*: Harris Hip Score, *THA* total hip arthroplasty

**Table 2:** Surgical data

Patient	Surgical approach	Acetabular cup (type and size)	Femoral stem (type and size)	Surgical time (min)	Orthopaedic length of stay (days)	Follow-up (months)
1	Lateral approach	Aldler Ortho Group B 3 36, Delta alumina Ceramic insert	Aldler Ortho Recta stem 3, Modula neck 12/14 0Y, Delta alumina Ceramic head 12/14 36 medium	88	8	121
2	Lateral approach	Zimmer Cup Continuum 48 GG, Biolox Delta Ceramic 32 mm	CLS Spotorno Stem 125 7, Biolox Delta Ceramic Head 32 35 L	139	5	45
	Lateral approach	Zimmer Cup Continuum 48 GG, Biolox Delta Ceramic 32 mm	CLS Spotorno Stem 125 7, Biolox Delta Ceramic Head 32 0 M	93	5	41
3	Lateral approach	Zimmer Cup Maxera press fit, 40 mm 50 mm	Stem Alloclassic SL 4 press fit, biolox delta ceramic XL 40+7	142	6	42
4	Anterior approach	Lima Cup Delta TT/one 54 mm, Delta polyethylene insert 36 mm	Lima Stem minima monolithic standard 8, Lima Biolock head, ceramic neck s 32 mm	230	7	40
5	Anterior approach	Zimmer Biomet acetabular cup 50, Delta ceramic insert 32	Zimmer Biomet GTS stem 3, ceramic head L 32	150	8	48
6	Anterior approach	Lima Cup Delta PF 62 mm, Delta ceramic insert 36 mm	Lima Minima S Lat 5, Delta ceramic head 36 mm, neck M	130	6	15
7	Posterolateral approach	Jump system Traser Permedica 44, Polyethylene insert 0°	Modular Stem EXACTA SM NR. 1, ceramic head 28 mm S, neck XS	100	9	32
8	Posterolateral approach	Zimmer Cup Continuum 44, Ceramic insert	Wagner Cone 125° nr 15. Ceramic head 28 M	110	15	26
9	Posterolateral approach	Zimmer Cup TM Tantalum + 2 screws, Polyethylene insert 28	Zimmer Cone 135° N 20, ceramic head BIOLOX 28 mm, medium neck	130	6	38
	Posterolateral approach	Jump system Traser Permedica 48, Polyethylene anti-dislocation insert 32	Stem EXACTA Permedica NR. 4, ceramic head 32 mm M, neck S	120	12	8
10	Posterolateral approach	Zimmer Cup TM Tantalum 50 + 2 screws, Polyethylene insert 28	Wagner stem nr 17, ceramic head BIOLOX 32 mm, medium neck	85	8	15

**Table 3:** Comparison of results based on the surgical approach

	Mean age	Surgical time (min)	Dislocation rate	Orthopaedic length of stay (days)
Anterior approach (*n* = 3)	15	170	0%	7
Lateral approach (*n* = 4)	16.3	109	0%	6
Posterolateral approach (*n* = 5)	18.6	115.5	40%	10

Clinical outcomes were assessed comparing the Harris Hip Score (HHS) administered before THA and at the last follow-up (Table 1). Serial anteroposterior and axial radiographs of the operated joints were reviewed to assess the position of the implant and possible signs of loosening and wear.

Statistical analysis was performed using GraphPad Prism 6.0 software (GraphPad Software Inc., La Jolla, CA, USA). Student’s *t* test was applied to assess any statistical difference between pre- and postoperative clinical findings, with a *p* < 0.05 considered statistically significant).

## Results

Ten consecutive patients (12 hips) affected by JHOA, aged between 14 and 20 years old, were reviewed. Among the conditions causing JHOA, SCFE affected five patients, LCPD two, and DDH two (both hips in a single patient) (Table 1). Patient no. 2 developed bilateral osteonecrosis of femoral head after being treated for acute lymphoblastic leukaemia with chemotherapy and glucocorticoids for 2 years.

**Fig. 1:**
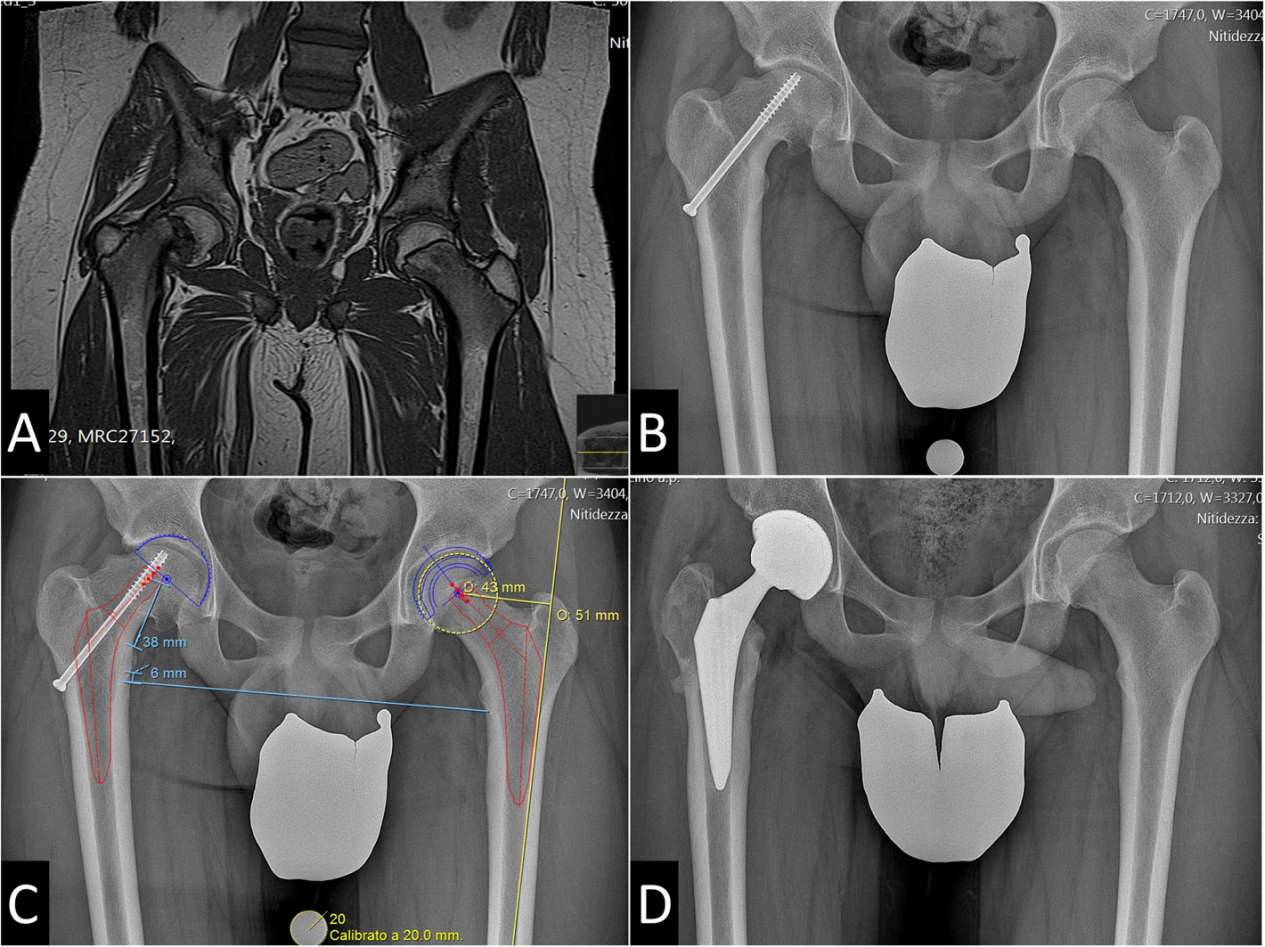
**a** Plain radiographs of a 15-year-old male with right SCFE (patient 5). **b** Screw fixation of the right hip. **c** Preoperative planning. **d** Postoperative radiograph after right THA using anterior approach

**Fig. 2:**
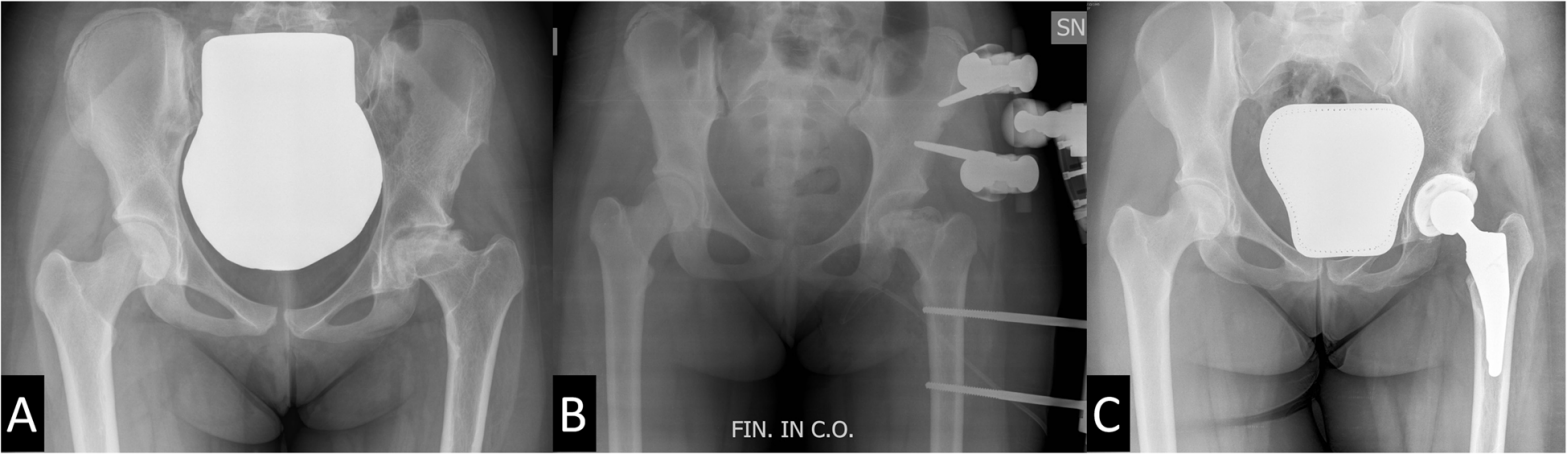
**a** Plain radiographs of a 19-year-old female with LCPD (patient 7). **b** Arthrodiatasis of the left hip with external fixator. **c** Postoperative radiograph after left THA using posterolateral approach

Most patients had undergone other forms of hip surgery prior to THA: 3 Dunn procedures, 2 screw fixation, one Chiari’s pelvic osteotomy and one arthrodiatasis (Table 1, Figs. 1 and 2). All the operated patients underwent hardware removal before THA surgery. The average age at the time of THA was 17.0 years (range 14–20 years).

The THA procedure was performed through a direct lateral approach in 4 patients, tissue-sparing direct anterior approach in 3 patients and a posterolateral approach in 5 patients. Overall, the average surgical time was 126 min (range 85–230 min) and was longer for the direct anterior approach, even though statistical significance was not reached. The average length of stay in the orthopaedic unit was 8.1 days (range 5–15 days). All patients were then transferred to the rehabilitation unit (mean length of stay 15.9 days, range 12–22 days). The average duration of follow-up was 3.3 years (range 0.7–10.1 years).

Comparing the preoperative and postoperative HHS, the score improved significantly in all patients (mean preoperative HHS 36.1 versus mean postoperative HHS 94.0, *p* < 0.01). There were no complications except for one transient femoral nerve palsy (patient no. 1), resolved without any further treatment in 2 months and two hip dislocations in the posterolateral approach group. Patient no.7 dislocated in the first postoperative day and underwent closed reduction under sedation on the same day. Patient no. 9 underwent right hip dislocation in the second postoperative day and the following day underwent open reduction and substitution of the acetabular insert with a hooded anti-dislocation polyethylene insert.

## Discussion

Total hip arthroplasty has become a safe routine procedure in middle-aged and elderly population with predictably good outcomes [33, 34]. The treatment of paediatric hip disorders secondary to SCFE, LCPD, femoral head necrosis, and DDH still presents a challenge. Data on the long-term follow-up in adolescents and high-demand young adults is limited. Compared to the adult population, such patients experience more complications and earlier revision from aseptic loosening of either the acetabular, more common or the femoral component [35, 36]. For these reasons, we reserve THA to those patients who have very serious limitations in everyday life from hip pain and loss of function and after conservative treatments failure [6]. THA in severe hip diseases in young individuals is technically difficult, as the proximal femoral geometry and acetabular orientation may be aberrant [28]. A careful preoperative planning in paediatric hip disease is crucial to obtaining good outcomes; the choice of the most suitable implant must be suited to the anatomy of each individual patient. In this respect, modular implants may help surgeons to restore femoral version and offset [37].

The medical literature reports increased operative time and complication rates when the removal of previously implanted hardware is performed at the same time of THA [38]. This supports routine implant removal in children with a high likelihood of future THA [39]. In most of these patients, the choice of the appropriate surgical approach requires an understanding of the local anatomy to optimize joint visualization [28]. A longer surgical time seems to be related (although in the present series, statistical significance was not reached) to the use of the anterior approach for THA.

The anterior and lateral approach may have a lower rate of dislocations in the immediate postoperative period compared to the posterolateral approach. In our patients, it is difficult to understand whether this different dislocation rate resulted purely from the different surgical approach or the greater average age of the posterolateral approach cohort (Table 2). In fact, the older the patient, the worse the hip deformity from the original condition [40] (Table 3). On the other hand, preservation of the posterior soft tissues may also explain the lower dislocation rate observed with the lateral and anterior approaches compared to the posterior one. A meta-analysis reported an 8 times greater dislocation rate when soft tissue repair was not performed in adults operated using the posterior approach [41]. Adjusting femoral anteversion while respecting acetabular anteversion in THA in paediatric hip disorders could effectively prevent dislocation, enhance the reliability of cup-bone osteointegration and reduce the risk of hip iliopsoas pain after THA. In our opinion, minimal technical modifications on these patients allow to obtain better results. In paediatric hip diseases, careful reconstruction of the posterior capsule and external rotators may be fundamental to decrease the risk of postoperative dislocation when using the posterior approach [41]. Furthermore, the only patient (patient 9) who underwent a THA revision with insert substitution after dislocation had been operated on for DDH. Attention should be paid during surgical planning especially for this subgroup of patients. In fact, instability is the fourth cause of THA failure in young patients, but the second cause of failure in THA performed for DDH, comparable in frequency for revision for acetabular loosening and wear [36].

In any case, THA clearly improves the HHS also in these young patients, and our results are in line with the most recent literature [42]. Nevertheless, the correct timing of this surgery remains unknown. We do not know whether it is better for a patient to undergo THA when serious functional limitations start or whether it is better to wait for symptoms to become severely disabling, forcing these patients to a lower quality of life for months or years before proposing THA. The patients reported in this case series had not undergone regenerative medicine attempts before THA. This reflects the severe osteoarthritic changes that all patients showed at presentation. Also, 4 patients had already undergone osteotomies. Osteotomies are still a good solution to gain time before THA. Nevertheless, as for regenerative medicine, they are contraindicated when severe osteoarthritic changes involve both the femoral head and the acetabulum, as was the case for the patients reported in the present investigation [43].

For less severe osteoarthritis, we consider two additional factors in the surgical decision-making process. The first are patients’ symptoms. Severe functional impairment during adolescence could impact negatively on the emotional sphere, and THA allows a more rapid and long-term recovery than osteotomies. The second factor is the severity of osteoarthritis. An osteotomy performed on an already osteoarthritic bone would at best result in only a short-term improvement of symptoms and could represent a complicating factor for the future THA surgery [39]. Furthermore, regardless of whether the osteotomy fixation hardware is removed at another surgery, or during the index THA, thus prolonging the surgical times, the risk of subsequent periprosthetic joint infection is theoretically increased [44]. In general, we prefer to remove the metalwork, if previous surgery has been undertaken, well before the arthroplasty is performed [39]. Further studies are necessary to answer these legitimate questions and assess the safest surgical approach for these young patients.

We acknowledge that this study has several limitations. The main limitation is the limited sample size and the different follow-up times; given the early diagnosis and the successful treatment of the less severe presentations of the hip developmental diseases, JHOA has currently become relatively rare [6]. We suspect that multicentre studies will be necessary to collect enough data on these challenging patients and randomized controlled trials will be difficult to perform.

## Conclusions

Hip replacement in carefully selected young patients is safe and reliable and should be considered after conservative management has failed to restore hip function. In our cohort, THA demonstrated maintenance of improved clinical outcomes at 3.3 years from the index procedure. The right timing for THA remains unknown, although strongly conditioned by the quality of life of these patients.

Careful preoperative planning is crucial, as technical modifications are sometimes mandatory to adapt the THA procedure to the abnormal anatomy of each patient.

## Data Availability

All data generated or analysed during this study are included in this published article.

## Abbreviations

DDH: Developmental dysplasia of the hip
SCFE: Slipped capital femoral epiphysis
JHOA: Juvenile hip osteoarthritis
AVN: avascular necrosis
THA: Total hip arthroplasty
HHS: Harris Hip Score
